# Random telegraph noise from resonant tunnelling at low temperatures

**DOI:** 10.1038/s41598-017-18579-1

**Published:** 2018-01-10

**Authors:** Zuo Li, Moïse Sotto, Fayong Liu, Muhammad Khaled Husain, Hiroyuki Yoshimoto, Yoshitaka Sasago, Digh Hisamoto, Isao Tomita, Yoshishige Tsuchiya, Shinichi Saito

**Affiliations:** 1 Sustainable Electronics Technologies, Department of Electronics and Computer Science, Faculty of Physical Science and Engineering, University of Southampton, Southampton, UK; 2 Research and Development Group, Hitachi, Ltd., 1-280 Higashikoigakubo, Kokubunji, Tokyo 185-8601 Japan

## Abstract

The Random Telegraph Noise (RTN) in an advanced Metal-Oxide-Semiconductor Field-Effect Transistor (MOSFET) is considered to be triggered by just one electron or one hole, and its importance is recognised upon the aggressive scaling. However, the detailed nature of the charge trap remains to be investigated due to the difficulty to find out the exact device, which shows the RTN feature over statistical variations. Here, we show the RTN can be observed from virtually all devices at low temperatures, and provide a methodology to enable a systematic way to identify the bias conditions to observe the RTN. We found that the RTN was observed at the verge of the Coulomb blockade in the stability diagram of a parasitic Single-Hole-Transistor (SHT), and we have successfully identified the locations of the charge traps by measuring the bias dependence of the RTN.

## Introduction

Charge traps in advanced Silicon (Si) Metal-Oxide-Semiconductor Field-Effect Transistors (MOSFETs) have been used for many important applications^1^ in semiconductor industries. For example, a Metal-Oxide-Nitride-Oxide-Semiconductor (MONOS) memory device^2,3^ has an advantage on a long retention time at high temperatures, which is suitable for an integrated microprocessor in a vehicle. An interface trap is also useful to extend the current plateau in a single electron pump^4^ at low temperatures, which is a promising candidate for a redefinition of ampere to establish a new current standard based on an elementary charge in quantum metrology. On the other hand, charge traps also affect reliability problems^5,6^, such as Random Telegraph Noise (RTN)^7–11^ and Negative Bias Temperature Instabilities (NBTI)^12^, causing failures of Static Random Access Memory (SRAM) cells and degradations of long term performance^13,14^. In particular, the impact of RTN is getting more important with the scaling^15–17^, since the variations^18–20^ in an atomic level can affect the drain current in sub-20 nm MOSFETs.

RTN is coming from the carrier trapping and de-trapping processes through charge traps at the gate insulator/Si interface, which are intrinsic quantum processes^21,22^. Quantum effects are not negligible in advanced MOSFETs, since the gate insulator is as thin as 1 nm^23^, and the direct tunnelling currents from the channel into the traps are expected due to enhanced coupling^24^. Previously, the most of the works on RTN were based on measurements at room temperatures^7–11,25,26^. At room temperatures, the thermal fluctuation significantly affect the carrier dynamics^27^, and it was difficult to identify the quantum energy levels of charge traps. By measuring the MOSFETs at low temperatures, it is easier to observe various quantum effects^28^.

In our previous study, we have found current peaks at low temperatures in advanced Si MOSFETs^29^, but the mechanism of the peaks was not elucidated. In this paper, we characterised the peaks in time domain, and found that the current peaks are related to RTN. The bias conditions to observe the current peaks have been clarified from the stability diagram of the associated with a parasitic Single-Hole-Transistor (SHT) at low temperatures. We also studied the bias dependence of RTN to identify the possible origin of charge traps.

## Current Peaks in the Stability Diagram

**Figure 1:**
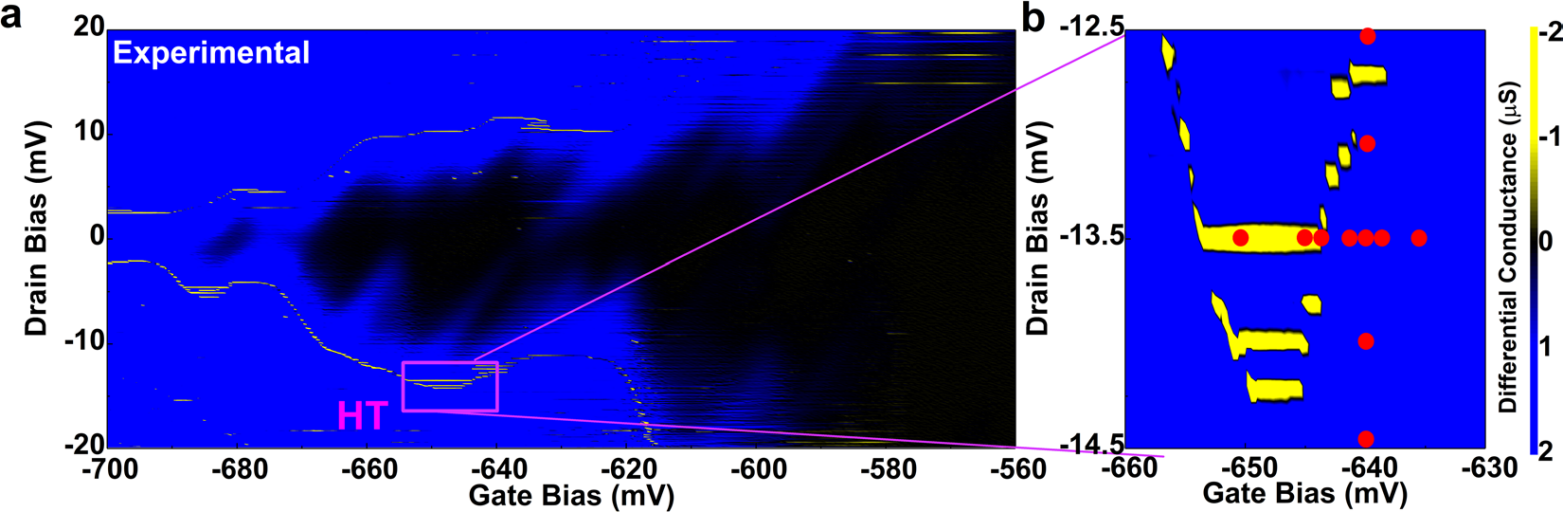
Bias condition to observe current peaks. (**a**) Shows the stability diagram of the *p* MOSFET at 2 K. The regime where current peaks can be observed (negative differential conductance) was shown. (**b**) Shows the extended view of HT region and the bias conditions where RTNs were measured.

The device was measured at 2 K, and the fundamental characteristics are shown in the supplementary information. From the stability diagram, open diamonds were observed in the stability diagram of the device, as shown in Fig. 1(a), which implies that several quantum dots in series were responsible for I_d_ in the subthreshold regime. The quantum dots might be coming from the remote surface roughness coming from Poly-Si grains^30,31^. At the edge of Coulomb diamonds, we have observed current peaks in the stability diagram, which were previously reported in our studies on MOSFETs at low temperatures^29^. The regime where current peaks can be observed formed a border at the edge of Coulomb diamonds. Particularly, the density of current peaks was much higher at some narrow bias conditions. We mark one of them as HT. The extended view of stability diagram in HT regime is shown in Fig. 1(b).

## Quantum Probabilities of the Trap States

**Figure 2:**
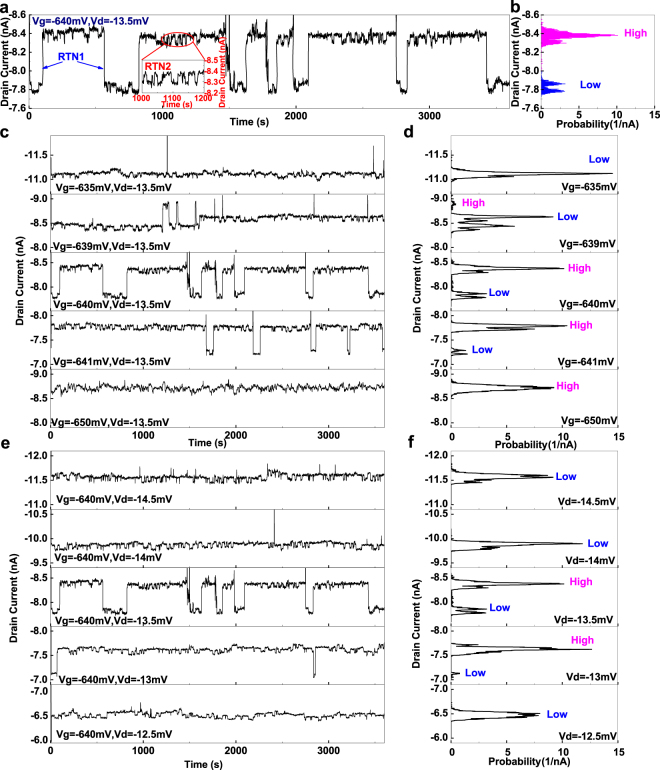
Time domain characteristics of *I*_d_. (**a**) Shows the time domain characteristics of *I*_d_ measured at *V*_g_ of −640 mV and *V*_d_ of −13.5 mV. RTN1 and RTN2 are marked. (**b**) Shows the probability distribution of current versus its value. The state ‘High’ and ‘Low’ regarding RTN1 is marked. (**c**) Shows the dependence of RTN on *V*_g_ if *V*_d_ is biased at −13.5 mV, and (**d**) Shows the probability distribution of current accordingly. (**e**) Shows the dependence of RTN on *V*_d_ if *V*_g_ is biased at −640 mV, and (**f**) shows the probability distribution of current accordingly.

We measured the time domain characteristics of *I*_d_ at the bias conditions near the current peaks, and observed RTN. One example of the measurement results, which was measured at *V*_g_ of −640 mV and *V*_d_ of −13.5 mV, is shown in Fig. 2(a). Two types of RTN could be identified, which were named as RTN1 and RTN2, as shown in Fig. 2(a). RTN1 has larger amplitude and longer average switching time, while RTN2 has smaller amplitude and shorter average switching time. The long switching time and high amplitude imply that the trap corresponding to RTN1 is a deep oxide trap, while RTN2 might come from the shallow interface trap in the SiON/substrate interface. We could roughly estimate the amplitude of RTN1 by estimating the number of carriers inside the channel^32–34^, *N*_0_ = *k*_B_ *TC*_gate_/*e* ^2^ = *k*_B_ *T* ${\varepsilon }_{{\rm{ox}}}$ ${\varepsilon }_{0}$ *WL*/*t*_eq_ *e* ^2^ = 11.6, where *k*_B_ is Boltzmann constant, e is the value of elementary charge, ${\varepsilon }_{{\rm{ox}}}$ is the dielectric constant of SiO_2_, ${\varepsilon }_{0}$ is the permittivity in vacuum, W is the width of the channel, L is the length of the channel and ${t}_{{\rm{e}}{\rm{q}}}$ is the capacitive equivalent thickness of SiON layer^35–37^. Then the relative amplitude of RTN1 can be estimated as Δ*I*/*I* = 1/*N*_0_ = 8.6%, which was roughly in agreement with the experimental data.

In order to study the statistics of *I*_d_, we analysed the frequency with which we observed *I*_d_ at certain ranges, with the minimum step of 4pA, which was determined by the systematic noise of the system in a bandwidth of 5 Hz, as shown in Fig. 2(b). This is the quantum mechanical probability finding the system under a certain current state, *P*(*I*_d_), which reveals the information about the wave function of the traps, *ψ*(*I*_d_), as

1 $$P({I}_{{\rm{d}}})={|\psi ({I}_{{\rm{d}}})|}^{2}\mathrm{.}$$

At higher temperatures, RTN1 could not be observed, which is shown in the supplementary data. This is because the number of carriers significantly increased at higher temperatures^38,39^. As a result, the relative amplitude of RTN dropped, and will be hidden in the 1/f noise.

In order to study the bias dependence of RTN1 and RTN2, the time domain characteristics of *I*_d_ were measured at the bias conditions marked as red points in Fig. 1(c). The dependence of the time domain characteristics on *V*_g_ is shown in Fig. 2(c). The corresponding *P*(*I*_d_) at different bias conditions is shown in Fig. 2(d). RTN1 was only observed in a narrow window of *V*_g_, between −635 mV and −650 mV. We observed that the current was more likely to be in the high current state if we increased |*V*_g_|. This showed the charge trap shifted from the unoccupied state to the occupied state as we changed *V*_g_. It is also in agreement with the *I*_d_ − *V*_g_ characteristics near the HT regime as shown in supplementary data.

The *V*_d_ dependence on time domain characteristics is shown in Fig. 2(e), and the corresponding *P*(*I*_d_) at different bias conditions is shown in Fig. 2(f). The high current state regarding RTN1 was only observed at a narrow window of *V*_d_, between −13 mV and −13.5 mV. This narrow bias window, along with the fact that the current peaks were observed at the edges of the Coulomb diamonds, implies that the RTN1 has originated from resonant tunnelling^40^.

## Nature of the trap

**Figure 3:**
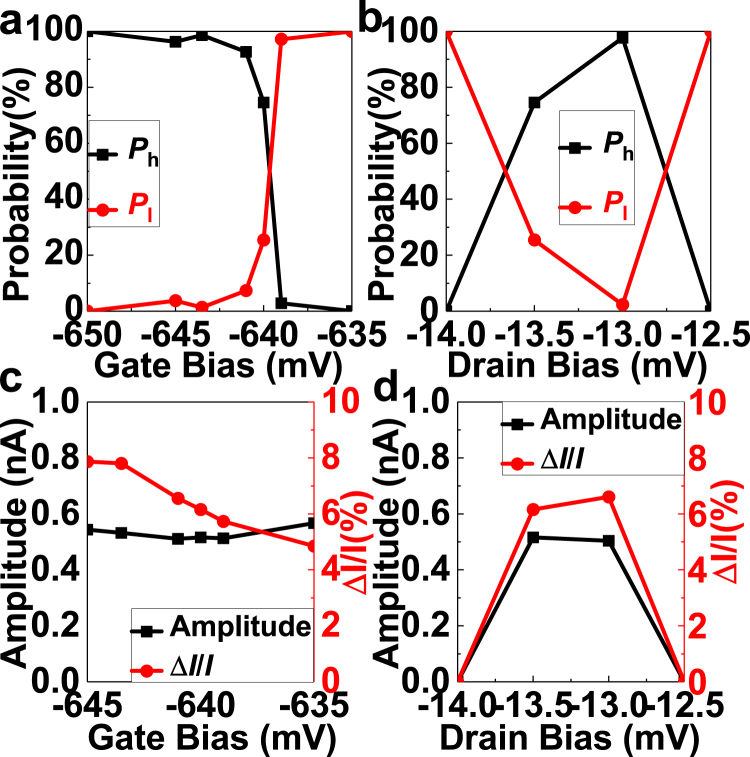
The bias dependence of RTN1. (**a**) and (**b**) Shows the dependence of *P*_h_ and *P*_1_ on *V*_g_ and *V*_d_, respectively. (**c**) and (**d**) Shows the dependence of RTN1 amplitude on *V*_g_ and *V*_d_, respectively.

We will invesigate on the probability of finding *I*_d_ at the high current State (*P*_h_) or the low current state (*P*_1_) shown in Fig. 2(b) to identify the nature of the charge trap. The way to extract *P*_h_ and *P*_1_ is shown in the method. The dependence of *P*_h_ and *P*_1_ on *V*_g_ and *V*_d_ are shown in Fig. 3(a) and (b), respectively.

From Fig. 3(a), *I*_d_ was more likely to be in the high current state if |*V*_g_| was large, and a sharp transition of the preferred *I*_d_ state can be observed near *V*_g_ of −640 mV. This sharp transition showed that the occupancy of the trap, which is revealed from *P*_h_ and *P*_1_, was modulated by *V*_g_. This implies the charge trap corresponding to RTN1 is located in the SiON layer. The high current state was only observable at *V*_d_ of −13 mV and −13.5 mV accroding to Fig. 3(b), which implies the presence of resonant tunnelling in the channel due to the narrow bias window of *V*_d_. The *V*_g_ and *V*_d_ dependence of RTN1 amplitude were shown in Fig. 3(c) and (d), respectively. We can see that the amplitude of RTN1 almost has no dependence on both *V*_g_ and *V*_d_. Since the dependence of *I*_d_ on *V*_g_ is non-linear, as shown in supplementary information, RTN1 is unlikely to have come from the impact of the shift of threshold voltage caused by carrier trapping/de-trapping. Otherwise, if the shift of threshold voltage was constant, the amplitude of RTN1 will have dependence on *V*_g_ and *V*_d_, which was not what we observed in Fig. 3(c). We could use a similar method to investigate the characteristics of RTN2. The dependence of RTN2 on biases was complex, which implies RTN2 came from a shallow trap near SiON/substrate interface. The dependence of RTN2 on biases is shown in Supplementary information.

**Figure 4:**
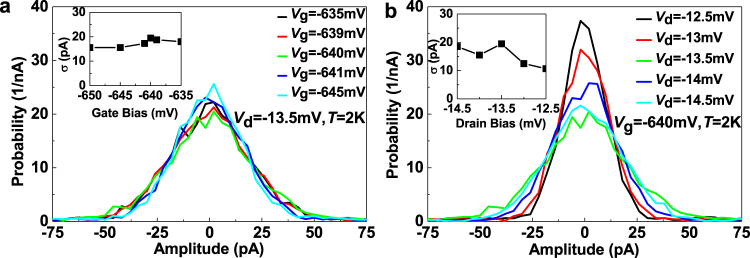
The dependence of experimental parameters on wavefunction broadening. (**a**) and (**b**) Shows the dependence of wave function broadening on *V*_g_ and *V*_d_, respectively. The *V*_g_ and *V*_d_ dependence on standard deviation of the corresponding Gaussian wave function, *σ*, is shown in subfigures inside (**a**) and (**b**), respectively.

The position of the single hole is determined by the wavefunction of single hole in the charge trap. Different positions of the single hole in the charge trap show different impact on the *I*_d_. Therefore, the wavefunction of the single hole in this charge trap resulted in an extra noise in *I*_d_. In order to understand the wavefunction of the single hole as shown in Fig. 2(d) and (f), we must study its bias dependence. We will study the noise coming from wavefunction in order to understand its dependence on biases. The differential current, Δ*I*_d_, is defined as

2 $${\rm{\Delta }}{I}_{{\rm{d}}}={I}_{{\rm{d}}}(t+\mathrm{1)}-{I}_{{\rm{d}}}(t),$$

where t is time with the unit of second. The dependence of its probability distribution on *V*_g_ and *V*_d_ are shown in Fig. 4(a) and (b), respectively. The standard deviation of Δ*I*_d_ was extracted by fitting the distribution of Δ*I*_d_ with the Gaussian distribution function.

The probability distribution of Δ*I*_d_ had very weak dependence on *V*_g_, as shown in Fig. 4(a), while it showed more significant dependence on *V*_d_, as shown in Fig. 4(b). Since the change of *I*_d_ is similar between Fig. 4(a) and (b), the fact that Δ*I*_d_ shows more significant dependence on *V*_d_ implies that the coupling between the quantum dot and energy level created by charge trap was mainly modulated by *V*_d_. We observe that the standard deviation of probability distribution showed a peak value if *V*_d_ was at −13.5 mV, as shown in Fig. 4(b). This reveals that the wavefunction of the single hole in the channel became the broadest at this bias condition, which implies that the strongest correlation between two energy levels in the channel at this bias condition. This is also in agreement with the assumption of resonant tunnelling. The resonant level is likely to have originated from the charge traps. The Johnson noise^41,42^ of this system was calculated to be ~20 fA/$\sqrt{Hz}$ and the background noise of the system was ~4pA in a bandwidth of 5 Hz, which were both much smaller than the noise coming from wavefunction.

## Two Traps RTN

**Figure 5:**
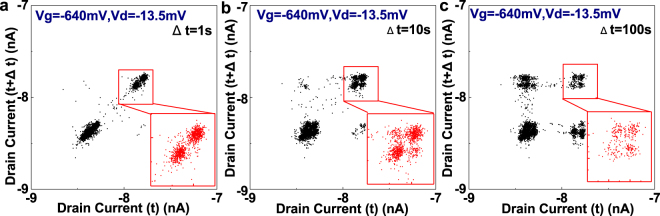
Fractal nature of the charge traps. The lag plot of *I*_d_ with different time lag shows fracture nature of charge traps. (**a**–**c**) Shows the correlation behaviour of *I*_d_ if time lag was 1 s, 10 s and 100 s, respectively.

We could use lag plots^43^ to study the correlation behaviour of *I*_d_ and the fractal nature of the two charge traps^44^. The lag plot of *I*_d_, with the time lag, Δ*t*, of 1 s, 10 s and 100 s, were shown in Fig. 5(a),(b) and (c) respectively.

When Δ*t* was 1 s, we could observe strong correlation behaviour, as shown in Fig. 5(a). The fractal shape of the lag plot is diagonal, which reveals the strong positive autocorrelation behaviour of *I*_d_ in this time scale. When Δ*t* was increased to 10 s, the shape of lag plot remained diagonal, but become rectangular within each small area, as shown in Fig. 5(b). This reveals the lost of *I*_d_ correlation behaviour regarding RTN2. When Δ*t* was further increased to 100 s, the fractal shape of lag plot became rectangular in both large and small area, as shown in Fig. 5(c). This reveals that the correlation behaviour of *I*_d_ almost disappeared in this time scale.

## Discussion

In the previous sections, we discussed about the characteristics of charge traps. Based on the experimental data, we found that the RTN1 is coming from the opening/closing of a resonant level in the device channel, and the carrier trapping/de-trapping process have originated from the tunnelling process between the Poly-Si gate and charge trap in the SiON layer. The lag plot clearly shows the correlation behaviour and fractal nature of the two charge traps. We also observed the crossover between RTN and 1/f noise, which was previously reported to be observed in magnetic nanodot system^45^, as shown in the Supplementary information.

**Figure 6:**
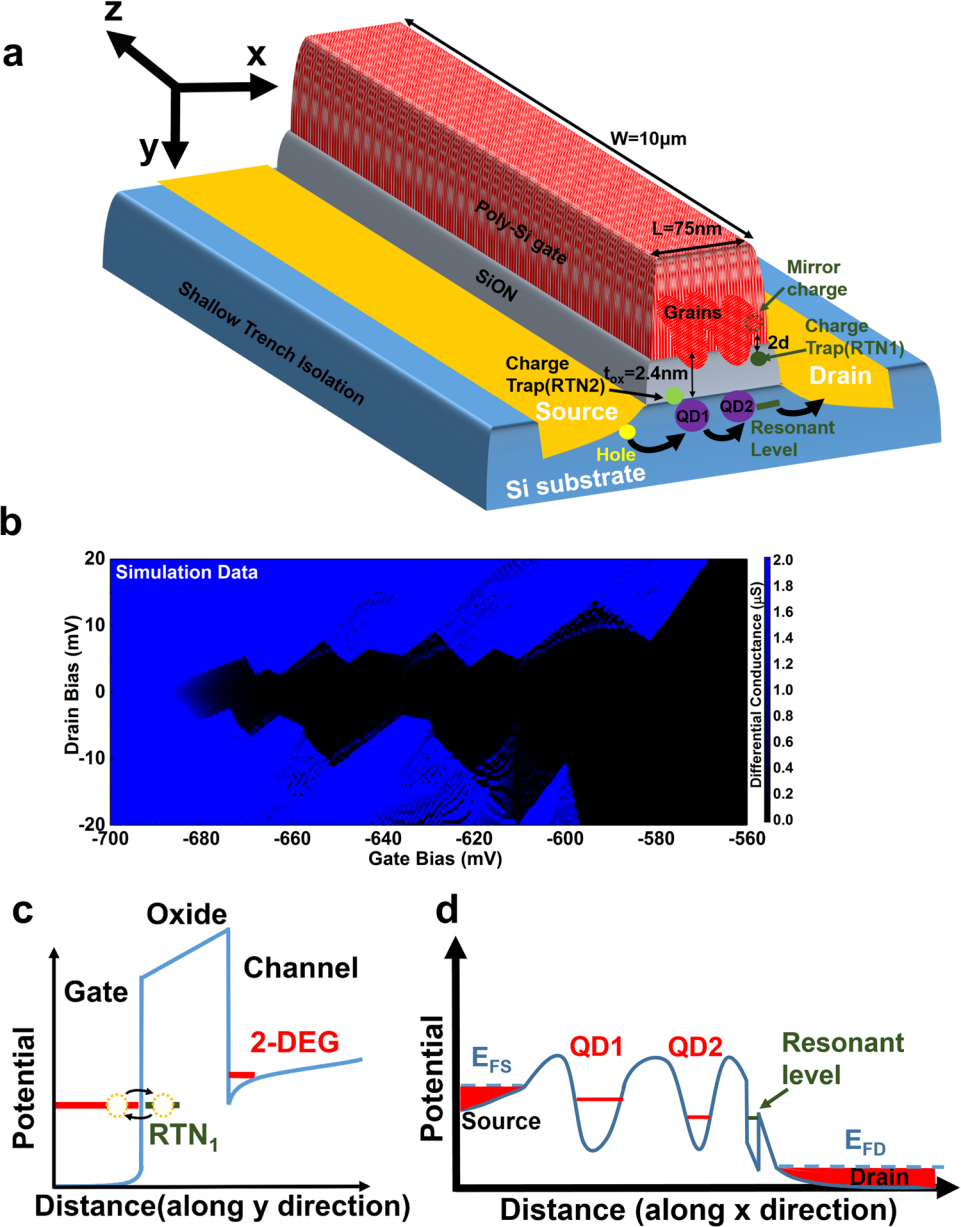
The physical model for the RTN. (**a**) Shows the schematic physical model of the device. Series quantum dots are assumed to be responsible for the drain current, and the resonant level was formed by the trap 1, presumably a Boron ion. (**b**) Shows the simulated stability diagram based on the series quantum dots model. (**c**) Shows the schematic potential diagram across the oxide layer associated with the charge trapping and de-trapping process. (**d**) Shows the schematic diagram of the potential across the channel.

Based on the information shown above, we could establish a physical model to describe the RTN1, as shown in Fig. 6. A schematic 3-D diagram of the device and its physical model is shown in Fig. 6(a). Two quantum dots in series, marked as QD1 and QD2, were responsible for the drain current. The series quantum dots have presumably originated from the remote surface roughness caused by Poly-Si grains. We simulated the stability diagram of the series quantum dots system based on the master equations^46,47^, which is explained in detail in method part. The parameters are summarised in the supplementary information. The simulated stability diagram is shown in Fig. 6(b), which is roughly in agreement with the experimental data obtained in Fig. 1(a). The simulation result of stability diagram for individual QD1 and QD2 have been summarised in Supplementary information.

The trap corresponding to RTN1 is located in the SiON layer. Considering the *V*_g_ dependence, the charge trapping/de-trapping process has likely originated from the tunnelling of carriers between the poly-Si gate and the trap inside the SiON, as shown in Fig. 6(c). If |*V*_g_| is decreased, the tunnelling barrier between the hole in the poly-Si gate and the energy level in the gate oxide becomes lower, and the trap was easier to be occupied. This corresponds to the trend observed in Fig. 3(a).

The different current states can be explained by the opening/closing of the resonant level^48–50^ in the channel created by the charge trap. Due to the strong *V*_d_ dependence, the resonant level should be close to the drain reservoir. We address the resonant level to be located in the tunnelling barrier between QD2 and the drain reservoir. As a result, the resonant level is strongly coupled with QD2. Due to the relatively much larger distance between the resonant level and QD1, the coupling between the resonant level and QD1 is much weaker than the coupling between the resonant level and QD2, and therefore can be neglected.

A schematic diagram of the potential profile across the channel is shown in Fig. 6(d). If no single hole occupies the charge trap, it was not neutral. Since the Poly-Si gate behaves like a metal, the trap generates mirror charge in the Poly-Si gate. As a result, the trap and the corresponding mirror charge forms an electric dipole. The electric dipole therefore forms a potenetial well for the single hole in the channel. Under certain bias conditions, the energy level inside this potential well is aligned with the energy level in the QD2. Under that circumstance, resonant tunnelling is observed and the tunnelling barrier between QD2 and drain reservoir is made more transparent. As a result, *I*_d_ will increase, therefore we can observe the high current state. If the charge trap is occupied by a single hole, the charge trap will become neutral, and no resonant level will be found in the channel. Under this condition, the tunnelling barrier therefore becomes less transparent, and we can only observe the low current state. This model, which is based on resonant tunnelling, could explain the reason for the narrow *V*_g_ and *V*_d_ bias condition to observe RTN. Since the resonant level is in the channel, it is therefore more significantly modulated by *V*_d_. Besides, the transmission coefficient and wavefunction were mainly influenced by the *V*_d_, which explains the fact that the wavefunction was mainly affected by *V*_d_, as shown in Fig. 4.

We can estimate the position of charge trap based on the depth of the resonant energy level. From the simplicity point of view, since RTN1 is only observed in the HT regime at the edge of Coulomb diamond, the depth of the potential well, Δ*V*, is assumed to be 13.5 mV. We could estimate the distance between trap and Poly-Si/SiON interface, d, from

3 $$e{\rm{\Delta }}V=\frac{2{e}^{2}d}{4\pi {\varepsilon }_{{\rm{ox}}}{\varepsilon }_{0}{{t}_{{\rm{eff}}}}^{2}}\mathrm{.}$$

From Equation (3), we can roughly estimate d to be ~0.2 nm. This is on the same magnitude with the lattice constant of SiON. This estimation should be correct in magnitude, and is in agreement assuming that the charge trap is located on the top side of SiON. As a result, we think a charge trap located near the Poly-Si/SiON interface, which was presumbaly a boron ion coming from the ion implantation process, is responsible for the resonant level in the channel.

In conclusion, we successfully demonstrate that we could identify the nature of the trap by measuring the RTN and investigating its bias dependence at low temperatures. We could estimate the position of the trap from the experimental data. Our research demonstrate a way to study the characteristics of charge trap systematically, and will pave the way for scientists to understand the detailed nature of the RTN.

## Methods

### Sample and experiments

The device we measured was a standard bulk-Si *p*-type MOSFET (*p* MOSFET), fabricated by a standard 65nm-node technology. The channel of the *p* MOSFET was 10 μm wide and 75 nm long. Wide-channel devices were chosen to increase the chance to find charge traps. The gate was made of highly-doped poly-crystalline silicon (Poly-Si). The gate oxide was made of SiON, with the equivalent oxide thickness of 2.4 nm. The *p* MOSFET was wire-bonded with Aluminium wire onto a chip carrier. Then the *p* MOSFET was put into a cryostat, with the maximum capability to control temperatures down to 2 K. The *I*_d_ was measured by a B1500A with high resolution current module. The value of current at each bias was obtained after averaging over 10^5^ sampling taken with the duration of 2 μs for each point. The background noise was less than 4pA, in a bandwidth of 5 Hz.

### Data analysation

The probability to observe high current state and low current state in Figs 2 and 3 are determined by fitting the experimental data with Gaussian distribution functions. The amplitude of RTN is determined by the difference of the peaks. The probability of each state is determined by integrating the corresponding probability distribution function over the current. For example, if the probability distribution function corresponding to the high current state in Fig. 2 is *p*_h_(*I*_d_), and the probability distribution function corresponding to the low current state in Fig. 2 is *p*_1_(*I*_d_), *P*_h_ and *P*_1_ are determined from

4 $${P}_{{\rm{h}}}=\frac{{\int }_{-\infty }^{\infty }{p}_{{\rm{h}}}({I}_{{\rm{d}}})d{I}_{{\rm{d}}}}{{\int }_{-\infty }^{\infty }{p}_{{\rm{h}}}({I}_{{\rm{d}}})d{I}_{{\rm{d}}}+{\int }_{-\infty }^{\infty }{p}_{{\rm{l}}}({I}_{{\rm{d}}})d{I}_{{\rm{d}}}},$$

and

5 $${P}_{{\rm{l}}}=\frac{{\int }_{-\infty }^{\infty }{p}_{{\rm{l}}}({I}_{{\rm{d}}})d{I}_{{\rm{d}}}}{{\int }_{-\infty }^{\infty }{p}_{{\rm{h}}}({I}_{{\rm{d}}})d{I}_{{\rm{d}}}+{\int }_{-\infty }^{\infty }{p}_{{\rm{l}}}({I}_{{\rm{d}}})d{I}_{{\rm{d}}}},$$

which correspond to the shaded areas (magenta and blue, respectively) in Fig. 2(b).

### Simulation of Quantum Dots

In semiconductor quantum dots, the single particle energy spacing is comparable to the charging energy. Therefore, the Hamiltonian of the system does not simply depend on the charging energy, and the effect of single particle spacing must be considered. In order to simulate the quantum dots in a simple way, we used mesoscopic capacitor model, with different effective coupling capacitances in different hole states, as an approximation.

Assuming the gate capacitance, drain capacitance, and source capacitance when n holes occupy the quantum dots are *C*_g_(n), *C*_d_(n) and *C*_s_ (n) respectively. Considering the circumstance when one less hole occupies the quantum dot, the change in free energy when a hole tunnels out through the electrode drain, ${\rm{\Delta }}{F}_{{\rm{d}}}^{-}(n)$, or tunnels out through the electrode source, ${\rm{\Delta }}{F}_{{\rm{s}}}^{+}(n)$, can be expressed as

6 $${\rm{\Delta }}{F}_{{\rm{d}}}^{-}(n)={F}_{{\rm{d}}}(n-\mathrm{1)}-{F}_{{\rm{d}}}(n)=-\frac{(n-\mathrm{1/2)}{e}^{2}-e[({C}_{{\rm{s}}}(n)+{C}_{{\rm{g}}}(n)){V}_{{\rm{d}}}-{C}_{{\rm{g}}}(n){V}_{{\rm{g}}}]}{{C}_{{\rm{\Sigma }}}(n)},$$

and

7 $${\rm{\Delta }}{F}_{{\rm{s}}}^{+}(n)={F}_{{\rm{s}}}(n-\mathrm{1)}-{F}_{{\rm{s}}}(n)=\frac{(n-\mathrm{1/2)}{e}^{2}+e[{C}_{{\rm{d}}}(n){V}_{{\rm{d}}}+{C}_{{\rm{g}}}(n){V}_{{\rm{g}}}]}{{C}_{{\rm{\Sigma }}}(n)}\mathrm{.}$$

We could easily obtain the expression of ${\rm{\Delta }}{F}_{{\rm{d}}}^{+}(n)={F}_{{\rm{d}}}(n+\mathrm{1)}-{F}_{{\rm{d}}}(n)$, and ${\rm{\Delta }}{F}_{{\rm{s}}}^{-}(n)={F}_{{\rm{s}}}(n+\mathrm{1)}-{F}_{{\rm{s}}}(n)$ from Eqs (6) and (7). Therefore, using Fermi’s golden rule, the tunnelling rate through the drain and source can be expressed as

8 $${{\rm{\Gamma }}}_{{\rm{d}}}^{\pm }(n)=\frac{1}{{R}_{d}{e}^{2}}[-\frac{{\rm{\Delta }}{F}_{{\rm{d}}}^{\pm }(n)}{1-\exp (\frac{{\rm{\Delta }}{F}_{{\rm{d}}}^{\pm }(n)}{{k}_{{\rm{B}}}T})}],$$

and

9 $${{\rm{\Gamma }}}_{{\rm{s}}}^{\pm }(n)=\frac{1}{{R}_{s}{e}^{2}}[-\frac{{\rm{\Delta }}{F}_{{\rm{s}}}^{\pm }(n)}{1-\exp (\frac{{\rm{\Delta }}{F}_{{\rm{s}}}^{\pm }(n)}{{k}_{{\rm{B}}}T})}]\mathrm{.}$$

The master equation can be expressed as

10 $$\frac{\partial p(n,t)}{\partial t}=p(n+\mathrm{1)}[{{\rm{\Gamma }}}_{{\rm{s}}}^{+}(n+\mathrm{1)}+{{\rm{\Gamma }}}_{{\rm{d}}}^{-}(n+\mathrm{1)}]-p(n)[{{\rm{\Gamma }}}_{{\rm{s}}}^{-}(n)+{{\rm{\Gamma }}}_{{\rm{d}}}^{+}(n)]\mathrm{.}$$

In the steady state, the probability is not assosiated with time, so

11 $$\frac{\partial p(n,t)}{\partial t}=0.$$

The drain current is therefore expressed as

12 $$I=e{\sum }_{n=-\infty }^{n=\infty }p(n)[{{\rm{\Gamma }}}_{{\rm{s}}}^{+}(n)-{{\rm{\Gamma }}}_{{\rm{s}}}^{-}(n)]\mathrm{.}$$

We solve the equations listed above using extracted parameters to simulate the characteristics of drain current and differential conductance in series quantum dots system. The effect of inversion layer when *V*_g_ is near the threshold voltage is considered in the simulation.

## Electronic supplementary material

Supplementary Information
