# Supplementary material for: Random telegraph noise from resonant tunnelling at low temperatures
Source: Sci Rep. 2018 Jan 10;8:250. doi: 10.1038/s41598-017-18579-1 (PMC5762822; doi:10.1038/s41598-017-18579-1)
Supplement: Supplementary file 1 — Supplementary Information [file 41598_2017_18579_MOESM1_ESM.pdf]

Supplementary Information: Random Telegraph Noise from resonant tunnelling at low temperatures

Zuo Li[1], Moïse Sotto[1], Fayong Liu[1], Muhammad Khaled Husain[1], Hiroyuki Yoshimoto[2], Yoshitaka Sasago[2], Digh Hisamoto[2], Isao Tomita[1], Yoshishige Tsuchiya[1], and Shinichi Saito[1,*]

[1]Nano Research Group, Department of Electronics and Computer Science, Faculty of Physical Science and Engineering, University of Southampton, UK.
[2]Research and Development Group, Hitachi, Ltd., 1-280 Higashikoigakubo, Kokubunji, Tokyo 185-8601, Japan.
[*]S.Saito@soton.ac.uk

Single Hole Transistor Characteristics

We have measured the drain current ($I_{\rm d}$) of p-type MOSFET (Methods) at 300K, 150K, and 5K, respectively, at the drain bias ($V_{\rm d}$) of -50mV under the application of the gate bias ($V_{\rm g}$) from 0V to -1V, as shown in Fig. 1(a). The threshold voltage were -0.76V, -0.64V, and -0.54V at 2K, 150K, and 300K respectively. The sub-threshold slope was 80.0mV/decade at 300K, reduced to 48.4mV/decade at 150K, and became 9.8mV/decade at 2K.

We extracted the coupling capacitances from the experimental data, and used them as parameters in the simulation. In our simulation model, the capacitances for each hole state in QD1 and QD2 are different. Therefore, the slope for each edge of Coulomb diamonds in the stability diagram are different, which results in their different size, shape and asymmetry. The opening of the inversion channel in MOSFET is considered in the simulation.

From the extracted parameters, the coupling capacitances increased as $|V_{\rm g}|$ was increased. The simulation of stability diagram for QD1 and QD2 are shown in Fig. 1(b) and Fig. 1(c), respectively. The effect of inversion layer when $V_{\rm g}$ is near the threshold voltage is considered in the simulation. The names of the hole States are marked in the stability diagram.

Table 1: Extracted coupling capacitances of single-hole states in QD1.

State	$C_{\rm g}$(aF)	$C_{\rm d}$(aF)	$C_{\rm s}$(aF)
H0(QD1)	0.90	0.85	0.06
H1(QD1)	1.92	1.40	3.64
H2(QD1)	2.63	1.93	4.99
H3(QD1)	3.28	2.39	6.22
H4(QD1)	3.86	6.00	14.0

Table 2: Extracted coupling capacitances of single-hole states in QD2.

State	$C_{\rm g}$(aF)	$C_{\rm d}$(aF)	$C_{\rm s}$(aF)
H0(QD2)	0.73	0.71	1.94
H1(QD2)	1.77	2.43	4.73
H2(QD2)	2.46	2.43	5.81

We estimated the physical diameter of the quantum dot to be about 20.7nm for QD1 and 16.5nm for QD2.

Transport Characteristics

The $I_{\rm d}$-$V_{\rm g}$ and $I_{\rm d}$-$V_{\rm d}$ curves showed the information about the preferred state of $I_{\rm d}$ at different bias conditions, as shown in Fig. 2. The curves were extracted from the stability diagram in the main article. The actual measurement of the stability diagram was not performed on the same day as we measured the RTN characteristics. As a result, carriers might be trapped in some unexpected extremely long-life charge traps, resulting slight shift of bias condition to observe RTN1 most frequently. In the $I_{\rm d}$-$V_{\rm d}$ characteristics, we could identify the current peaks that were observable at the small windows of bias condition, as shown in Fig. 2(b). From Fig. 2(a), we could clearly address that the current is more likely to be in high current state if we increased $|V_{\rm g}|$ within the bias range we investigated.

Temperature Dependence of RTN1

The time domain characteristics of I_d at V_g of -640mV and V_d of -13.5mV were measured at 2K, 10K, 20K and 30K respectively, as shown in Fig. 3. RTN1 was not observed and hidden in the 1/f noise at temperatures higher than 10K, which was what we expected in the main manuscript.

Shallow Interface Trap

The RTN2 was investigated using the similar way as we investigated RTN1, as shown in Fig. 4. For the RTN2, we could observe three different current states, high, medium and low, as shown in Fig. 4(a), and we defined each state as h_2, m_2 and l_2, respectively. The level is determined by fitting the experimental data with Gaussian function, as shown in Fig. 4(b). The V_g and V_d of the $P(I_\mathrm{d})$ in state h_2, m_2 and l_2 are shown in Fig. 4(c) and (d), respectively. It can be found that the h2 state was only observed outside a certain bias window. For the probability, there is no obvious dependence on V_g and V_d. The amplitude shows a weak dependence on V_g and V_d. The complex dependence implies that the charge trap corresponding to RTN2 locates at SiON/substrate interface.

Correlation function

The Fourier transformation and correlation function of I_d were shown in Fig. 5(a) and Fig. 5(b), respectively. The peaks in correlation function reveals the time scale of RTN, and the symmetry of correlation function reveals that the process is a energy-conservative process. The 1/f dependence of power density was shown in Fig. 5(b). The crossover between RTN and 1/f noise was observed.

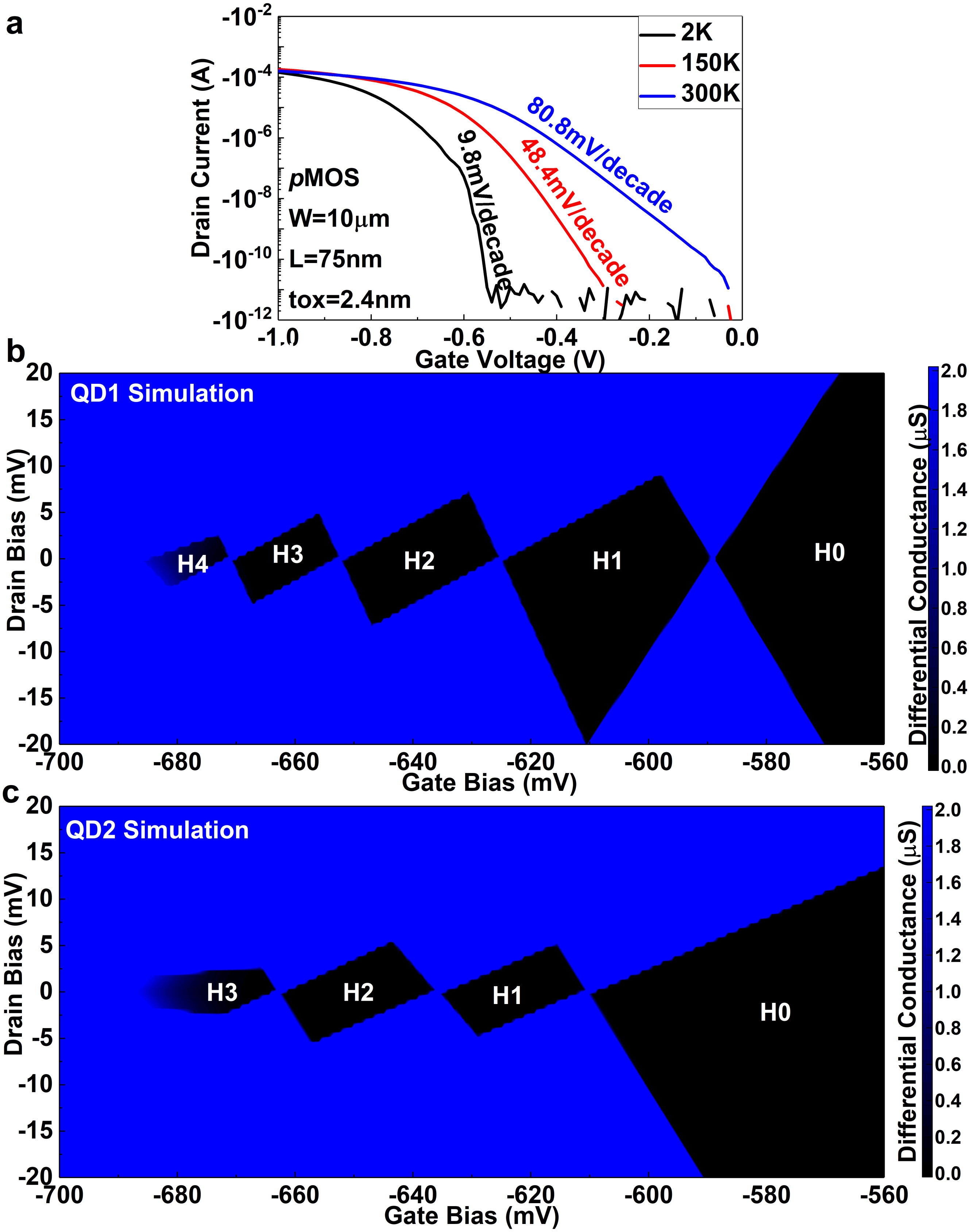

Figure 1: Characteristics of the pMOSFET. (a) shows the subthreshold characteristics of the pMOSFET at different temperatures. (b) shows the simulation result of stability diagram for QD1, and (c) shows the simulation result of stability diagram for QD2. All the hole states are marked on (b) and (c), respectively.

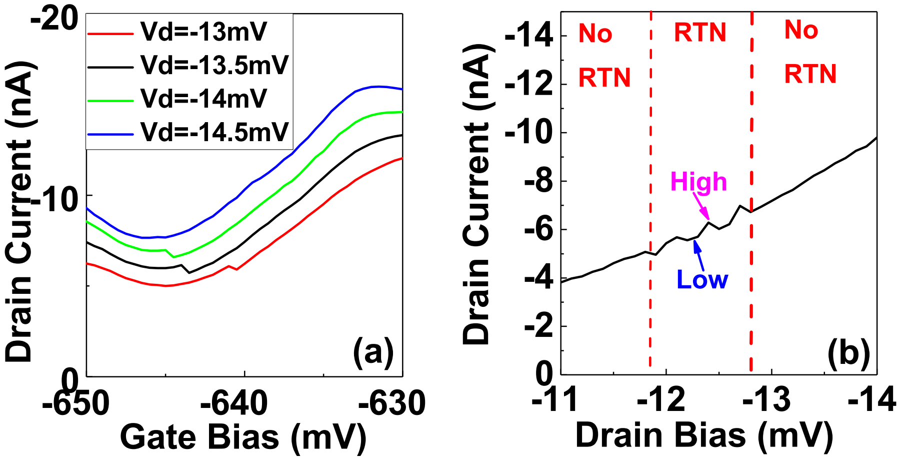

Figure 2: Transport characteristics of single hole. (a) shows the $I_{\rm d}$-$V_{\rm g}$ characteristics near the HT regime where we measured RTN. (b) shows the $I_{\rm d}$-$V_{\rm d}$ characteristics near the HT regime. Current peaks can be identified.

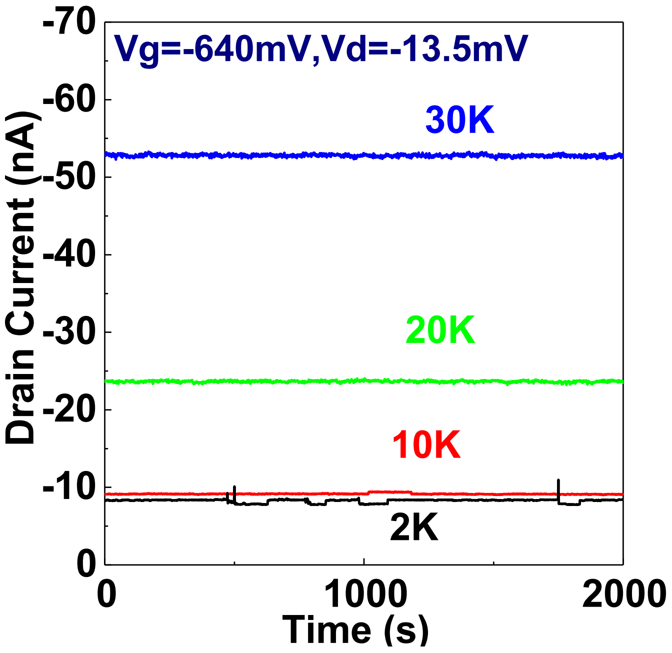

Figure 3: Temperature dependence of RTN1. The RTN1 was not observed at temperatures higher than 10K.

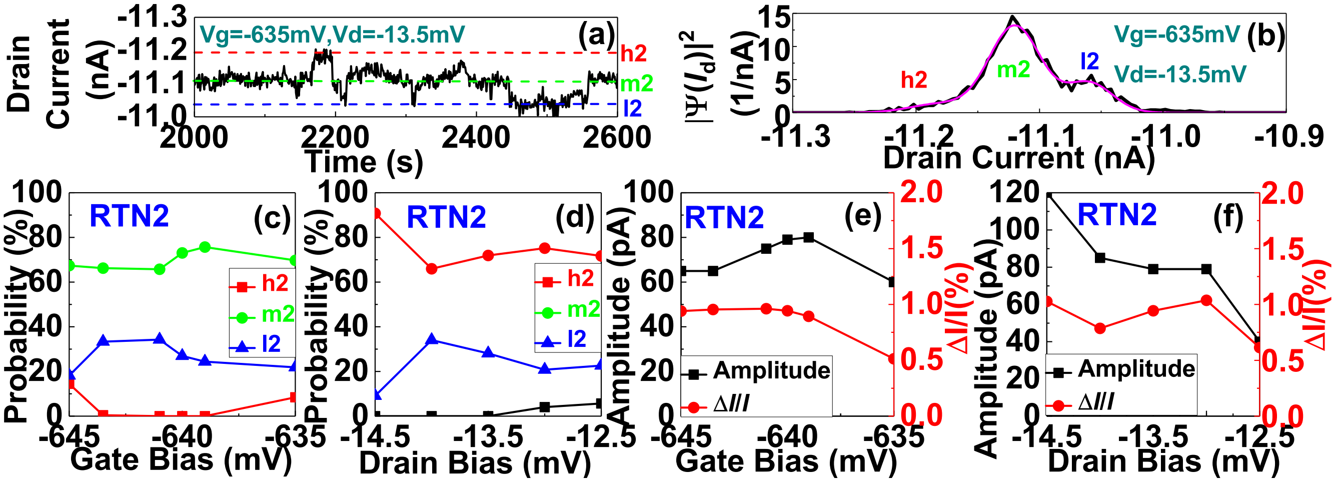

Figure 4: Investigation on RTN2. (a) shows the time domain characteristics of I_{d} at certain time range if V_{g} was biased at -635mV and V_{d} was biased at -13.5mV and (b) shows the corresponding wavefunction. The three current states, h2, m2 and l2, corresponding to RTN2, are marked in (b). (c) and (d) shows the dependence of probability on V_{g} and V_{d}, respectively. (e) and (f) shows the dependence of RTN2 amplitude on V_{g} and V_{d}, respectively.

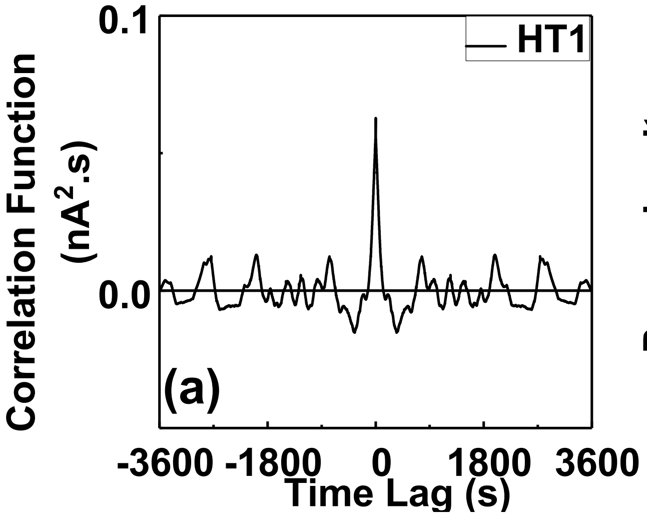

Figure 5: The correlation function of the I_{d}. The time domain characteristics were shown in (a) while (b) gives information about the power density obtained from Fourier transformation.
